# A Novel Approach to Sensorimotor Skill Acquisition Utilizing Sensory Substitution: A Driving Simulation Study

**DOI:** 10.1038/s41598-019-54324-6

**Published:** 2019-11-29

**Authors:** Sayako Ueda, Hiroyuki Sakai, Takatsune Kumada

**Affiliations:** 1 TOYOTA Collaboration Center, RIKEN Center for Brain Science, Wako, Japan; 2 Toyota Central R&D Laboratories, Inc., Nagakute, Japan; 3 Graduate School of Informatics, Kyoto University, Kyoto, Japan

**Keywords:** Sensorimotor processing, Human behaviour

## Abstract

The aim of this study is to demonstrate the potential of sensory substitution/augmentation (SS/A) techniques for driver assistance systems in a simulated driving environment. Using a group-comparison design, we examined lane-keeping skill acquisition in a driving simulator that can provide information regarding vehicle lateral position by changing the binaural balance of auditory white noise delivered to the driver. Consequently, lane-keeping accuracy was significantly degraded when the lower visual scene (proximal part of the road) was occluded, suggesting it conveyed critical visual information necessary for lane keeping. After 40 minutes of training with auditory cueing of vehicle lateral position, lane-keeping accuracy returned to the baseline (normal driving) level. This indicates that auditory cueing can compensate for the loss of visual information. Taken together, our data suggest that auditory cueing of vehicle lateral position is sufficient for lane-keeping skill acquisition and that SS/A techniques can potentially be used for the development of driver assistance systems, particularly for situations where immediate time-sensitive actions are required in response to rapidly changing sensory information. Although this study is the first to apply SS/A techniques to driver assistance, further studies are however required to establish the generalizability of the findings to real-world settings.

## Introduction

Generally, experiences in a given sensory modality are innately and persistently coupled with signals from a specific sensory organ (for example, the retina for vision). However, it is also an established fact that sensory mapping between sensory experiences and sensory organs can be very flexible. One of the most prominent examples of sensory remapping is the use of Braille by the blind. Braille constitutes a form of sensory substitution whereby blind persons can read Braille letters and numbers through tactile organs (i.e., fingertips). The use of a walking cane also constitutes a form of sensory substitution that allows blind individuals to navigate in outdoor environments. Furthermore, some blind persons can detect the location of surrounding objects via their auditory system by making use of echolocation cues (for a review, see Kolarik *et al*.^1^). A vast body of literature now shows that such sensory substitution skills in the blind are underpinned by enhanced plasticity within early sensory cortices^2–4^.

A number of sensory substitution devices have been proposed to facilitate sensory remapping in sensory-impaired individuals. These devices generally detect environmental information, normally conveyed by the impaired sensory organ, through electronic sensors and then convert this information into stimuli delivered to another intact sensory organ. For example, the vOICe^5^ sensory substitution system translates visual scenes recorded by a digital camera to soundscapes for blind individuals by converting elevation to pitch and brightness to loudness. Another sensory substitution device, EyeMusic^6^, further provides colour information by associating different colours with different musical instruments. The Tongue Display Unit^7^ translates visual images into electro-tactile stimulus patterns on the tongue. All these devices allow the user to receive information normally conveyed by the visual system via another intact sensory organ. Finally, the EyeCane^8^ detects the distance and angle from nearby obstacles using a built-in ultrasound sensor and converts the information into auditory and tactile stimuli, allowing blind individuals to navigate more comfortably in outdoor environments.

Sensory substitution can also be used in other settings that extend beyond the development of sensory prostheses for sensory-impaired individuals. It can be used in the development of tools designed to provide sensory augmentation in healthy individuals, which allows for the acquisition of novel or even non-innate sensory modalities and perceptual skills. For example, Nagel *et al*.^9^ proposed a waist belt-type vibration device that constantly indicates the magnetic north using a built-in digital compass. They demonstrated that individuals after six weeks of training could successfully learn to use the novel sense of magnetic orientation for navigation in outdoor environments. In another example, Konttinen *et al*.^10^ developed an auditory feedback system to augment the somatosensory information received during rifle shooting. The system provides real-time auditory feedback whose frequency corresponds to the aiming error measured during shooting. After four weeks of training using the system, participants showed better shooting performance when compared with participants who received the same amount of training without the auditory feedback.

Here, we propose a novel application of a sensory substitution/augmentation (SS/A) technique designed to assist drivers. Modern cars are loaded with various kinds of sensors that measure several features of the surrounding traffic environment, which are used to provide safety warning signals to drivers. To date, however, no assistant systems have been designed to augment the sensory information processing capacity of drivers. The use of SS/A techniques constitutes a promising avenue to successfully develop such systems. The most significant challenge for applying SS/A techniques to driver assistance, however, may be related to time constraints. Driving a car is an everyday sensorimotor skill that demands immediate actions in response to ever-changing contextual traffic situations, which require the real-time integration of multisensory (though mostly visual) information. Even in the simplest situation where one is driving a car on a road without any other cars or obstacles, the driver must keep the car in the correct lane by constantly using visual information from the lower visual scene (proximal part of the road) and must negotiate slight curves using visual information from the upper visual scene (distant part of the road)^11–13^ (see also the recent review by Lappi and Mole^14^ for a more in-depth discussion). To our knowledge, the online use of SS/A techniques under such high time pressure constraints has not been validated in previous applications.

In the current study, we thus aimed to demonstrate the potential application of SS/A techniques in the development of driver assistance systems. Specifically, using a group-comparison design, we examined lane-keeping skill acquisition in a driving simulator that can provide additional information regarding vehicle lateral position within a lane via the binaural balance of an auditory stimulus. If auditory cueing of vehicle lateral position can be used to compensate for occluded visual information that is necessary for lane-keeping (i.e., proximal part of the road), it is predicted that participants with SS/A assistance will show lane-keeping performance comparable to that of participants without SS/A assistance but that have access to the visual information necessary for lane keeping. It should be noted here that these experimental configurations were designed to test the applicability of SS/A techniques to driver assistance using a well-studied occlusion paradigm^12,13^, rather than to show whether the auditory cueing of vehicle lateral position is actually useful for lane-keeping in real traffic situations.

## Methods

### Participants

Forty-five naïve adults (25 females; 35 males) aged 18 to 30 years (M = 21.5 years; SD =1.8 years) participated in this study and received monetary compensation for their participation. All participants self-reported to have normal hearing and normal or corrected-to-normal vision. They were right-handed according to the Edinburgh Handedness Inventory^15^ and were ascertained to have normal visuomotor functions, as assessed by the Grooved pegboard test (Lafayette Instruments, Lafayette, IN). Written informed consent was obtained in accordance with a protocol approved by the RIKEN Research Ethics Committee [Wako3 28-17(4)].

### Driving simulator

**Figure 1:**
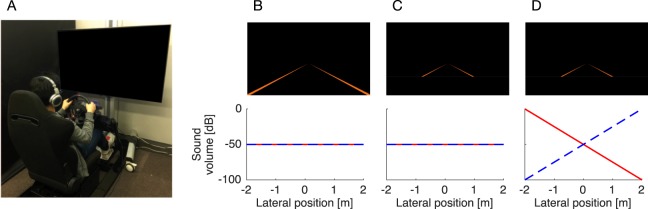
Apparatus and simulated driving task. Participants sat in the fixed-base driving simulator (**A**) and performed a lane keeping task in one of three training conditions. (**B**–**D**) In the normal driving (ND) training condition, lane marks on both sides of the road were entirely visible and no meaningful auditory information was provided. (**B**) In the visual occlusion (VO) training condition, the lower visual scene (proximal part of the road) was occluded and therefore visual cues that inform of vehicle lateral position were unavailable. (**C**) In the sensory substitution (SS) training condition, the proximal part of the road was also occluded and auditory cueing of vehicle lateral position was provided by changing the binaural balance of auditory white noise. Red solid lines and blue broken lines represent auditory stimulus intensity for the left and right ear, respectively.

Our driving simulator consisted of a fixed-base cockpit (GTD-SPECi, Rossomodello Co., Ltd., Tomioka, Japan), a force-feedback steering device (T500RS, Guillemot Corp., Carentoir, France), and a 60-inch LCD monitor (LC60XL10, SHARP Corp., Sakai, Japan) located in front of the cockpit (Fig. 1A). In this configuration, the monitor was viewed from a distance of approximately 115 cm. The custom-made software computed vehicle position and direction based on vehicle dynamics combined with steering inputs, and then displayed traffic scenes from a driver’s point of view on the monitor. The software could also occlude an arbitrary part of the visual scene, in addition to providing binaural auditory white noise (sampling frequency = 44.1 kHz) via headphones (HD 630VB, Sennheiser electronic GmbH & Co. KG, Wedemark, Germany). The simulation time step was 1/60 s.

### Procedure

The experiments were conducted in a quiet, dimly lit room. All participants were given a pretest session to familiarize themselves with the driving simulator. Specifically, they performed a lane-keeping task in which they were asked to keep the vehicle in the centre of a winding road course by using the steering wheel. In this task, the winding road course was defined by left and right lane marks that produced a lane width of 3.5 m (Fig. 1B) and was composed of alternating left and right bends with a random curvature between 1/180 to 1/80 m^−1^, interleaved with straight sections having a random length between 50 to 150 m. The vehicle automatically travelled with a constant speed of 80 km/h, which was empirically shown to be not too difficult for participants when negotiating curves in the present experimental set-up, and, therefore, no pedal operations were needed. In addition, no other road users were present and white noise with a constant level (−50 dB attenuated from the maximum level that was determined to be tolerable in a preliminary test to ensure participant comfort) was presented binaurally via headphones (Fig. 1B). The pretest session was composed of five one-minute trials with short breaks in between. After the pretest session, participants were randomly assigned to one of three experimental groups (15 participants per group): normal driving (ND) training condition, visual occlusion (VO) training condition, and sensory substitution (SS) training condition. The ND training group performed the lane-keeping task in an identical manner to that in the pretest session (Fig. 1B). For the VO training group, the lower visual scene (proximal part of the road) was occluded (Fig. 1C) to restrict the available visual cues necessary for the estimation of vehicle lateral position. For the SS training group, while the lower visual scene was also occluded, information regarding vehicle lateral position was provided via binaural auditory stimuli (Fig. 1D). More specifically, a leftward (rightward) deviation from the lane centre was signalled by increasing the sound level of the white noise in the left (right) ear and by decreasing it in the right (left) ear with a constant gain of 25 dB/m; in the lane centre, the sound level was equal in both ears (−50 dB). For all groups, the training session comprised 40 one-minute trials, with short breaks provided in between trials. Following the training session, participants completed a posttest session that was identical to the pretest session (i.e., five trials of the ND training condition). The entire experiment, including set-up and rest breaks, took approximately 60 min to complete.

### Data analysis

For each trial, driving performance was evaluated using two metrics^13^: *accuracy*, which was defined as the standard deviation of lateral position (SDLP) of the vehicle, and *stability*, which was defined as the mean maximum steering wheel velocity (SWV) during curve negotiation, averaged across all curves. For both performance metrics, lower values represented better performance. According to previous studies^12,13^, the accuracy metric reflects compensatory steering control that makes use of the visual information provided by the proximal part of the road, whereas the stability metric reflects anticipatory steering control that makes use of the information provided by the more distant part of the road. These findings demonstrate that the occlusion of the lower visual scene (proximal part of the road) compromises the accuracy but not the stability metric^12,13^.

The two performance metrics were separately submitted to two-way repeated measures analysis of variance (ANOVA) tests with the trial (first and last) as a repeated measure and with training condition (ND, VO, and SS) as a between-subjects factor. In addition, to examine whether the use of SS/A assistance for lane-keeping adversely affected ordinary driving skills acquired without assistance, the performance metrics were also compared between the last trial in the pretest session and the first trial in the posttest session for each training condition, using a two-way repeated measures ANOVA test with session (pretest and posttest) as a repeated measure and training condition (ND, VO, and SS) as a between-subjects factor. For all ANOVA tests, degrees of freedom were adjusted for sphericity using the Greenhouse-Geisser correction^16^. When a significant interaction was found, further simple main effect analysis and, if necessary, subsequent multiple comparisons using Shaffer’s modified sequentially rejective Bonferroni procedure^17^ were performed. A significance threshold was set at *P* < 0.05 for all tests.

### Ethics approval and consent to participate

All experiments were conducted in accordance with a protocol approved by the RIKEN Research Ethics Committee (Wako3 28-17(4)).

## Results

**Figure 2:**
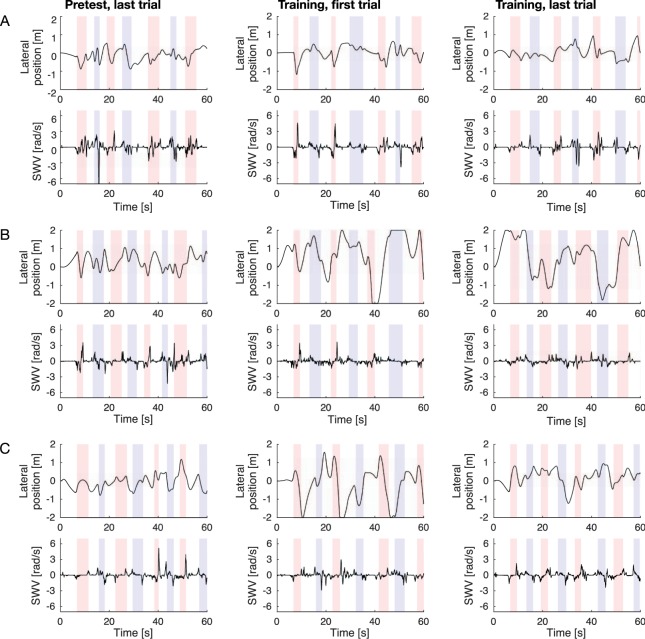
Examples of driving data. Each row corresponds respectively to a representative participant assigned to the ND (**A**), VO (**B**), or SS (**C**) training condition. From left to right, each column represents driving data during the last trial of the pretest session, and the first and last trials of the training session. For each panel, curve negotiation sections were colour-shaded (red, right-hand bends; blue, left-hand bends).

Figure 2 shows driving data obtained from a representative participant for each training condition. In the ND training condition (Fig. 2A), vehicle lateral position appeared to be relatively constant from the last trial of the pretest session to the last trial of the training session. In the VO training condition (Fig. 2B), as a result of the visual occlusion of the proximal part of the road, vehicle lateral position was found to be unstable throughout the entire training session compared with the last trial of the pretest session. In the SS training condition (Fig. 2C), increased variation in vehicle lateral position due to visual occlusion was also observed in the first trial of the training session (Fig. 2C). However, in the last trial of the training session, the participant again became able to control the vehicle more accurately, suggesting that auditory cueing was sufficient to enable proper vehicle control. In contrast, there seemed to be no remarkable changes in steering velocity throughout the trials.

**Figure 3:**
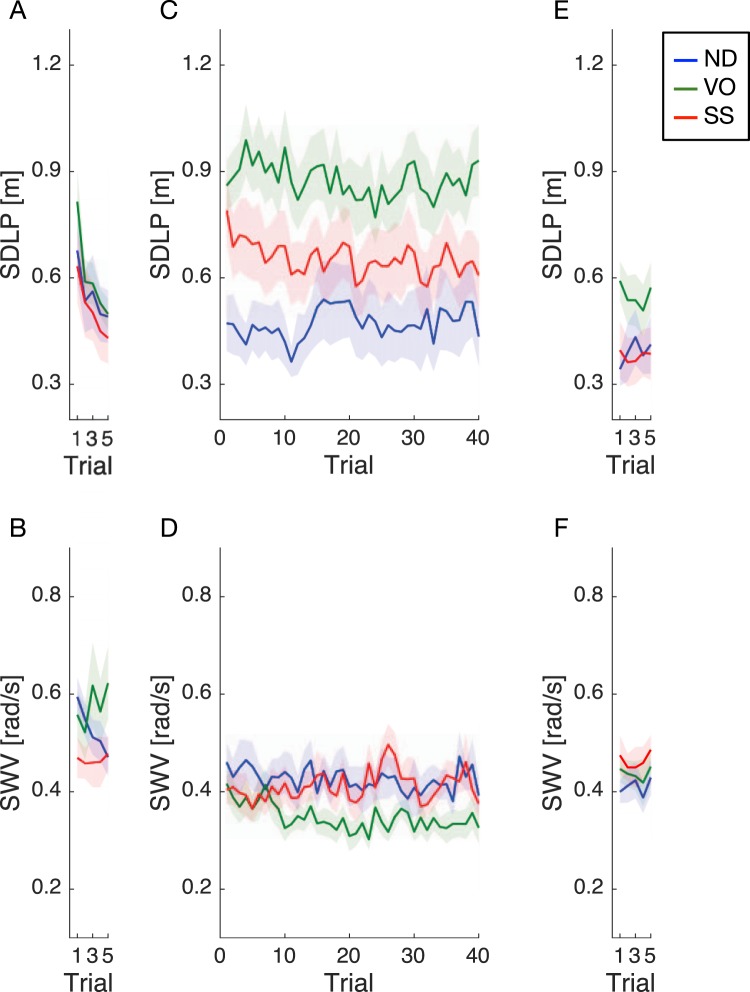
Temporal trajectory of each driving performance metric for the three training conditions. Driving performance was evaluated using two metrics: accuracy, which was defined as the standard deviation of lateral position (SDLP) of the vehicle, and stability, which was defined as the mean maximum steering wheel velocity (SWV) during curve negotiation, averaged across all curves. These metrics are illustrated for the following sessions: pretest (**A**, **B**), training (**C**, **D**), and posttest (**E**, **F**). Solid lines and shaded areas show the mean and standard error across participants, respectively. Different colours represent different training conditions.

Figure 3 shows the average temporal trajectory of both driving performance metrics for each training condition. In the pretest session, SDLP rapidly decreased for all conditions as trials were repeated (Fig. 3A). No systematic changes were observed for SWV (Fig. 3B). At the beginning of the training session, SDLP in the VO and SS training conditions increased due to, as expected, the loss of visual cues used for the estimation of vehicle lateral position (Fig. 3C). However, SDLP was found to decrease in the SS, but not in the VO training condition, as the training progressed and eventually reached a comparable level to that observed for the ND training condition by the end of the session (Fig. 3C). In contrast, SWV in the VO training condition tended to be lower than that observed in the ND and SS training conditions during the later trials of the training session (Fig. 3D). In the posttest session (Fig. 3E, F), both metrics were stable and similar in all training conditions, with the exception of somewhat larger SDLP values in the VO training condition.

**Figure 4:**
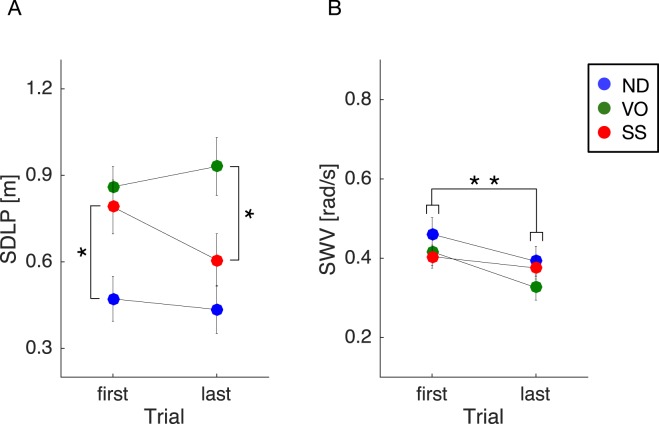
Training condition effects on driving performance metrics. Statistical comparisons revealed a significant training effect on SDLP, but only in the SS condition. (**A**) In contrast, there were no significant between-group training effects for SWV. (**B**) Error bars represent the standard error of the mean. Different colours represent different training conditions. Asterisks represent statistical differences (*P < 0.05; **P < 0.01).

In fact, an ANOVA on the SDLP values showed a significant interaction between trial and training condition [*F*(2, 42) = 5.23, *P* = 0.0093, *η*_*p*_^2^ = 0.20] (Fig. 4A). Subsequent post-hoc tests revealed a simple main effect of trial for the SS training condition [*F*(1, 14) = 15.94, *P* = 0.0013, *η*_*p*_^2^ = 0.53] and significant main effects of training condition for both the first [*F*(2, 42) = 6.42, *P* = 0.0037, *η*_*p*_^2^ = 0.23] and last [*F*(2, 42) = 7.85, *P* = 0.0013, *η*_*p*_^2^ = 0.27] trials. The SDLP values in the SS training condition, when compared to the ND training condition, were significantly larger during the first trial [*t*(42) = 2.53, *P* = 0.015] but equivalent during the last trial [*t*(42) = 1.11, *P* = 0.27]. The SDLP values in the SS training condition, when compared to the VO training condition, were not statistically different for the first trial [*t*(42) = 0.93, *P* = 0.36] but were significantly reduced for the last trial [*t*(42) = 2.74, *P* = 0.009]. In contrast, an ANOVA on SWV values from the training session revealed a significant main effect of trial [*F*(1, 42) = 9.31, *P* = 0.0039, *η*_*p*_^2^ = 0.18], no significant effect of training condition [*F*(2, 42) = 1.07, *P* = 0.35, *η*_*p*_^2^ = 0.05], and no significant interaction between trial and training condition [*F*(2, 42) = 0.3, *P* = 0.75, *η*_*p*_^2^ = 0.01] (Fig. 4B).

**Figure 5:**
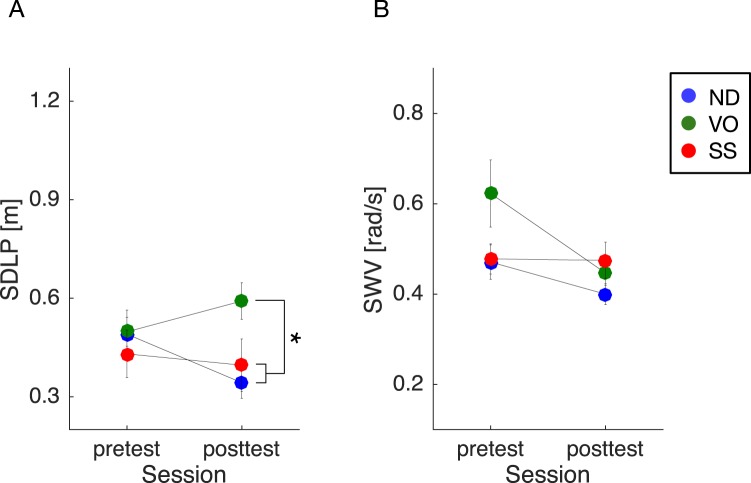
Pretest-posttest driving performance metric differences. Statistical comparisons revealed that in the posttest session, SDLP was significantly larger in the VO training condition than in both the ND and SS training sessions. (**A**) In contrast, there was no significant group difference for SWV. (**B**) Importantly, no obvious adverse interference effects of SS training were found for either SDLP or SWV during normal (vision-based) driving. Error bars represent the standard error of the mean. Different colours represent different training conditions. The asterisk represents a statistical difference (*P < 0.05).

Moreover, an ANOVA on SDLP pretest-posttest differences showed a significant interaction between session and training condition [*F*(2, 42) = 9.90, *P* < 0.001, *η*_*p*_^2^ = 0.32] (Fig. 5A). Subsequent post-hoc tests revealed no significant simple main effect of training condition for the pretest session [*F*(2, 42) = 0.33, *P* = 0.72, *η*_*p*_^2^ = 0.02], but did however reveal a significant simple main effect of training condition for the posttest session [*F*(2, 42) = 4.38, *P* = 0.018, *η*_*p*_^2^ = 0.17]. Multiple comparison tests further revealed that SDLP values in the posttest session were significantly larger for the VO training condition, compared with both the ND [*t*(42) = 2.81, *P* = 0.022] and SS [*t*(42) = 2.21, *P* = 0.033] training conditions. In contrast, an ANOVA on SWV pretest-posttest differences showed only a marginal interaction between session and training condition [*F*(2, 42) = 3.01, *P* = 0.06, *η*_*p*_^2^ = 0.13] (Fig. 5B). Importantly, no obvious adverse interference effects of SS training were found for either the SDLP or SWV metrics when compared to those obtained during normal (vision-based) driving.

To summarize, as hypothesized, lane-keeping accuracy was significantly degraded once the lower portion of the visual scene was occluded in the VO and SS training conditions. However, in the SS but not the VO training condition, vehicle lateral position stability was improved as the training progressed and the participants learned to use the auditory cueing. In addition, training with auditory cueing did not affect lane-keeping skills previously acquired based on intact visual information.

## Discussion

There is increasing evidence that sensory substitution devices can be used not only for sensory prostheses designed for sensory-impaired individuals but also for sensory augmentation purposes in healthy individuals. However, no study to date has examined whether such SS/A techniques can be used to develop driver assistance systems. Successful validation of SS/A-based driver assistance may extend the applicability of SS/A techniques to situations where immediate time-sensitive actions are required in response to rapidly changing sensory information.

In the present study, to demonstrate the potential applicability of SS/A techniques for the development of driver assistance systems, we examined the impact caused by auditory cueing of vehicle lateral position on lane-keeping skill acquisition in a simulated driving environment. We found that lane-keeping accuracy was initially degraded when the lower visual scene (proximal part of the road) was occluded. However, performance was found to recover when auditory cueing of vehicle lateral position was provided. This indicates, on the one hand, that the proximal part of the road is informative of vehicle lateral position and is critical for proper lane keeping, which is a robust replication of previous findings by Land and Horwood^12^ and Frissen and Mars^13^. On the other hand, and perhaps more importantly, these findings indicate that auditory cueing of vehicle lateral position provides sufficient information for proper lane keeping in the absence of the necessary visual information. Taken together, these results suggest that auditory cueing of vehicle lateral position is sufficient for lane-keeping skill acquisition and that SS/A techniques can be incorporated into driver assistance systems to support situation awareness, particularly in situations where rapid motor responses are required, such as requests to intervene in self-driving cars. This constitutes an important and novel finding, as the majority of prior SS/A studies examined performance in more static situations not requiring rapid responses^18–21^.

Contrary to accuracy, steering stability was not found to vary as a function of the different training conditions used in the present study. These findings are in agreement with a theoretical framework proposed by Donges^11^, which proposes that steering accuracy and stability are determined by two independent control processes. The first is a *compensatory process* that enables accurate steering control by using the visual information from the proximal part of the road (i.e., vehicle lateral position), and the second is an *anticipatory process* that enables stable steering control by using the visual information from the distant part of the road (i.e., curvature). In the present study, although the visualization of the proximal part of the road varied between training conditions, the distant part of the road was always visible for all training conditions. Therefore, the present results are consistent with the two-level theoretical framework of steering control, and also further suggest that auditory cueing of vehicle lateral position is important for the compensatory but not the anticipatory steering control process.

One concern regarding the application of SS/A techniques to healthy individuals was related to the possibility that the acquisition of newly acquired sensorimotor skills might interfere with an existing (intact) sensorimotor skill. In fact, it is well established that the consolidation of a motor skill is disrupted by a subsequently acquired motor skill, particularly when the latter is learned immediately after the former^22–24^. Nonetheless, the present study shows that training with a SS/A driver assistance system for lane-keeping did not adversely affect lane-keeping skills previously acquired. This indicates that sensory-motor skill may be robust against changing the source of sensory information insofar as it has sufficient accuracy.

Although our data indicate that SS/A techniques can be applied to the development of driver assistance technologies, its generalizability to real-world settings is still rather limited. One limiting factor in particular relates to the use of a driving simulator. Indeed, we tested the driver assistance feedback under optimal driving conditions where there were no pedestrians and oncoming cars. In real-world traffic environments, however, drivers are required to pay attention to various kinds of related and/or unrelated events on the road^25–27^. Such situations where attention must be carefully deployed might interfere with the use of substituted/augmented sensory information, or, conversely, the use of SS/A assistant system might interfere with the appropriate deployment of attention required for safe driving. In addition, we conducted all experiments under a constant vehicle speed. Thus, the generalizability of the current findings to different or varying speed conditions will need to be further investigated. Another limiting factor relates to the SS conversion rule used. In the present study, we examined auditory cueing of vehicle lateral position using a *natural* conversion rule, where approaching the left (right) lane mark is represented by an increase in the auditory stimulus intensity in the left (right) ear. We cannot conclude from the current findings whether SS/A driver assistance systems would also work when adopting a more artificial conversion rule that, for example, could translate vehicle lateral position to an auditory tone using a spatial-to-spectral conversion rule. Moreover, the usefulness of auditory cueing in real traffic environments may be somewhat limited because of potential acoustic interference from the radio or individuals conversing. Thus, the use of another sensory modality, e.g., haptic feedback^28,29^, could potentially prove to be useful for driver assistant systems using SS/A techniques.

Developing a better understanding of the neural substrate that underlies the processing of auditory cueing of vehicle lateral position is a relevant goal for future research. In the traditional view of sensory processing, sensory signals originating from a specific sensory organ are propagated toward a dedicated sensory cortex and, consequently, give rise to experiences in a unique sensory modality. This unimodal view of sensory processing suggests that auditory cueing of vehicle lateral position is primarily processed in the auditory cortex. However, recent findings have demonstrated that the prolonged use of SS/A can cause a reorganization of sensory cortices (for review, see Cecchetti *et al*.^30^). For example, it has been shown that after training with a visual-to-auditory substitution device, trained auditory stimuli activate cortical areas that are, in general, considered critical for the processing of visual features^31–34^. These findings highlight the multimodal nature of sensory cortices and suggest that, in fact, auditory cueing of vehicle lateral position might be processed in visual cortical areas that normally play a role in the extraction of vehicle lateral position from the proximal part of the road during normal driving.

In addition to exploring the underlying neural mechanisms, we envision several additional avenues of future research. For instance, a promising research direction is to expand the applicability of visual-to-auditory substitution for enhanced vehicle control. While in the present study we successfully conveyed the proximal part of the road with auditory cueing, future research could investigate the feasibility of additionally replacing the visual information from the distant part of the road with auditory cueing to determine if participants can accurately maintain lane-keeping with no visual information at all. Another direction of future research is to investigate the impact of auditory substitution on the driver’s eye-hand coordination strategy. It is well established that eye movement and steering behaviour are tightly related during curve negotiation; indeed, it has been shown that the driver fixates on the tangent point of an approaching curve about one second before steering the wheel^35,36^. It may thus be interesting to examine whether this eye-hand coordination is affected by visual occlusion and whether auditory substitution can compensate for the loss of visual information.

## Conclusions

We demonstrated that auditory cueing of vehicle lateral position enables accurate steering control, even in a situation where visual information normally necessary for lane-keeping is unavailable. This suggests, more generally, that SS/A techniques can be used in situations that demand immediate actions in response to the substituted sensory information. Further studies are required to establish the generalizability of the findings to more realistic real-world settings.

## References

[1] Kolarik AJ, Cirstea S, Pardhan S, Moore BC (2014). A summary of research investigating echolocation abilities of blind and sighted humans. Hear. Res..

[2] Uhl F, Franzen P, Lindinger G, Lang W, Deecke L (1991). On the functionality of the visually deprived occipital cortex in early blind persons. Neurosci. lett..

[3] Sadato N (1996). Activation of the primary visual cortex by Braille reading in blind subjects. Nature.

[4] Weeks R (2000). A positron emission tomographic study of auditory localization in the congenitally blind. J. Neurosci..

[5] Meijer PB (1992). An experimental system for auditory image representations. IEEE Trans. Biomed. Eng..

[6] Abboud S, Hanassy S, Levy-Tzedek S, Maidenbaum S, Amedi A (2014). EyeMusic: introducing a “visual” colorful experience for the blind using auditory sensory substitution. Restor. Neurol. Neurosci..

[7] Bach-y-Rita P, Kaczmarek KA, Tyler ME, Garcia-Lara J (1998). Form perception with a 49-point electrotactile stimulus array on the tongue: a technical note. J. Rehabil. Res. Dev..

[8] Maidenbaum S (2014). The “EyeCane”, a new electronic travel aid for the blind: Technology, behavior & swift learning. Restor. Neurol. Neurosci..

[9] Nagel SK, Carl C, Kringe T, Märtin R, König P (2005). Beyond sensory substitution—learning the sixth sense. J. Neural. Eng..

[10] Konttinen N, Mononen K, Viitasalo J, Mets T (2004). The effects of augmented auditory feedback on psychomotor skill learning in precision shooting. J. Sport. Exerc. Psychol..

[11] Donges E (1978). A two-level model of driver steering behavior. Hum. Factors.

[12] Land M, Horwood J (1995). Which parts of the road guide steering?. Nature.

[13] Frissen I, Mars F (2014). The effect of visual degradation on anticipatory and compensatory steering control. Q. J. Exp. Psychol..

[14] Lappi O, Mole C (2018). Visuomotor control, eye movements, and steering: A unified approach for incorporating feedback, feedforward, and internal models. Psychol. Bull..

[15] Oldfield RC (1971). The assessment and analysis of handedness: the Edinburgh inventory. Neuropsychologia.

[16] Geisser S, Greenhouse SW (1958). An extension of box’s results on the use of the *F* distribution in multivariate analysis. Ann. Math. Statist..

[17] Shaffer JP (1986). Modified sequentially rejective multiple test procedures. J. Amer. Stat. Assn..

[18] Auvray M, Hanneton S, O’Regan JK (2007). Learning to perceive with a visuo-auditory substitution system: localisation and object recognition with ‘The vOICe’. Perception.

[19] Proulx MJ, Stoerig P, Ludowig E, Knoll I (2008). Seeing ‘where’ through the ears: effects of learning by doing and long-term sensory deprivation on localization based on image to sound conversion. PLoS One.

[20] Haigh A, Brown DJ, Meijer P, Proulx MJ (2013). How well do you see what you hear? The acuity of visual-to-auditory sensory substitution. Front. Psychol..

[21] Stiles NR, Zheng Y, Shimojo S (2015). Length and orientation constancy learning in 2-dimensions with auditory sensory substitution: the importance of self-initiated movement. Front. Psychol..

[22] Brashers-Krug T, Shadmehr R, Bizzi E (1996). Consolidation in human motor memory. Nature.

[23] Walker MP, Brakefield T, Hobson JA, Stickgold R (2003). Dissociable stages of human memory consolidation and reconsolidation. Nature.

[24] Cohen DA, Robertson EM (2011). Preventing interference between different memory tasks. Nature Neurosci..

[25] Sakai H, Shin D, Kohama T, Uchiyama Y (2012). Attentional effects on gaze preference for salient loci in traffic scenes. Ergonomics.

[26] Underwood G, Chapman P, Brocklehurst N, Underwood J, Crundall D (2003). Visual attention while driving: sequences of eye fixations made by experienced and novice drivers. Ergonomics.

[27] He J, Becic E, Lee YC, McCarley JS (2011). Mind wandering behind the wheel: performance and oculomotor correlates. Hum. Factors.

[28] Abbink DA, Mulder M, Boer ER (2012). Haptic shared control: smoothly shifting control authority? *Cogn*. *Technol*. Work.

[29] Mulder M, Abbink DA, Boer ER (2012). Sharing control with haptics: Seamless driver support from manual to automatic control. Hum. Factors.

[30] Cecchetti L, Kupers R, Ptito M, Pietrini P, Ricciardi E (2016). Are supramodality and cross-modal plasticity the yin and yang of brain development? From blindness to rehabilitation. Front. Syst. Neurosci..

[31] Amedi A (2007). Shape conveyed by visual-to-auditory sensory substitution activates the lateral occipital complex. Nat. Neurosci..

[32] Striem-Amit E, Cohen L, Dehaene S, Amedi A (2012). Reading with sounds: sensory substitution selectively activates the visual word form area in the blind. Neuron.

[33] Striem-Amit E, Amedi A (2014). Visual cortex extrastriate body-selective area activation in congenitally blind people “seeing” by using sounds. Curr. Biol..

[34] Abboud S, Maidenbaum S, Dehaene S, Amedi A (2015). A number-form area in the blind. Nat. Commun..

[35] Land MF, Lee DN (1994). Where we look when we steer. Nature.

[36] Chattington M, Wilson M, Ashford D, Marple-Horvat DE (2007). Eye-steering coordination in natural driving. Exp. Brain Res..

